# Minimum Energy Conical Intersection Optimization Using DFT/MRCI(2)

**DOI:** 10.1021/acs.jctc.4c01489

**Published:** 2025-01-29

**Authors:** Tzu Yu Wang, Simon P. Neville, Michael S. Schuurman

**Affiliations:** † Department of Chemistry and Biomolecular Sciences, University of Ottawa, Ottawa K1N 6N5,Canada; ‡ National Research Council Canada, 100 Sussex Dr., Ottawa K1A 0R6, Canada

## Abstract

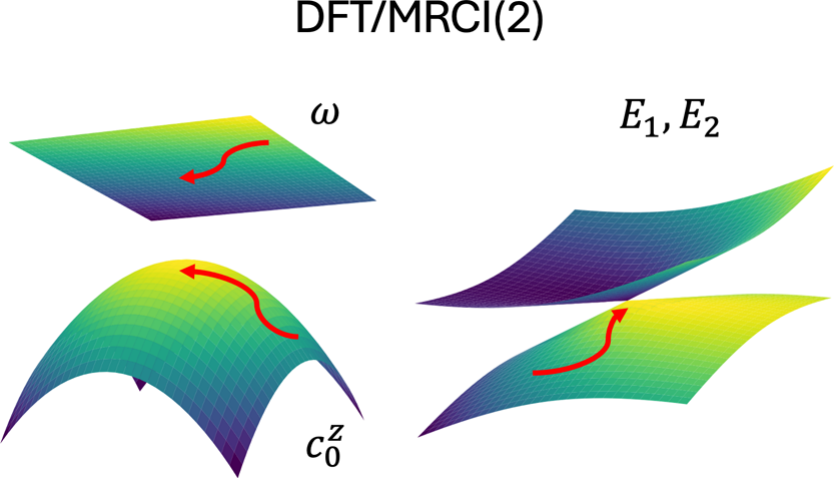

The combined density functional theory and multireference configuration interaction (DFT/MRCI) method is a semiempirical electronic structure approach that is both computationally efficient and has predictive accuracy for the calculation of electronic excited states and for the simulation of electronic spectroscopies. However, given that the reference space is generated via a selected-CI procedure, a challenge arises in the construction of smooth potential energy surfaces. To address this issue, we treat the local discontinuities that arise as noise within the Gaussian progress regression framework and learn the surfaces by explicitly incorporating and optimizing a white-noise kernel. The characteristic polynomial coefficient surfaces of the potential matrix, which are smooth functions of nuclear coordinates even at conical intersections, are learned in place of the adiabatic energies and are used to optimize the DFT/MRCI(2) minimum energy conical intersection geometries for representative intersection motifs in the molecules ethylene, butadiene, and fulvene. One consequence of explicitly treating the noise in the surfaces is that the energy difference cannot be made arbitrarily small at points of nominal intersection. Despite the limitations, however, we find the structures as well as the branching spaces to compare well with ab initio MRCI and conclude that this approach is a viable method to learn a smooth representation of DFT/MRCI(2) surfaces.

## 1 Introduction

The efficient generation of accurate molecular potential energy surfaces (PESs) is a perpetual challenge for theoretical chemistry. It is particularly difficult to reliably determine excited state PESs, which typically exhibit intersecting adiabatic surfaces. The accurate description of these electronic surfaces, as well as the reproduction of seams of conical intersection,^1^ are a requirement for dynamical studies of photoinitiated nonadiabatic processes.^2,3^ Generally, the required excited state PESs are generated using quantum chemistry methods, either via the parametrization of model potential functions or “on-the-fly”, as is common in trajectory simulations. The accuracy of these surfaces is strongly dependent on the level of electronic structure theory employed, and even different high levels of ab initio theory may yield qualitatively different excited state PESs. Electronic structure methods that are capable of furnishing accurate PESs over the range of electronic characters and nuclear configurations encountered in, e.g., organic photochemistry are typically characterized by an ability to describe electronic states for which a good zeroth-order description involves multiple electronic configurations. That is, those that are multireference in nature.^4^

Electronic structure methods that can simultaneously meet these requirements are generally based on restricted and complete active space self-consistent field (RASSCF/CASSCF)^5,6^ methods. Dynamic correlation may then be included by employing perturbation theory (as in the complete active space second-order perturbation theory, [CASPT2])^7,8^ or configuration interaction (multireference configuration interaction, [MRCI]).^9,10^ A consequence of the generality of active space methods is that they are typically computationally expensive. Furthermore, they generally require significant user input into the selection of the relevant active spaces, and the quality of the results is strongly dependent on how these spaces are chosen.

A class of methods that addresses some of these challenges are selected CI methods, which truncate the size of the Fock space by selecting only those configurations that are relevant to describe the states of interest. In particular, the combined density functional theory and MRCI (DFT/MRCI)^11,12^ method has been shown to be able to achieve an accuracy comparable to CASPT2 with reduced computational cost, as well good performance for various spectroscopic applications.^12−15^ This method employs a selected CI algorithm for the generation of the reference space, which when combined with DFT-specific corrections to the Hamiltonian matrix, results in highly compact wave function expansions. In addition, our group has developed a perturbative approximate variant of the parent DFT/MRCI method, which is termed DFT/MRCI(2)^14^ and drastically reduces the computational cost while maintaining the accuracy of the original method. In this work, we will primarily employ DFT/MRCI(2) and expect the results and conclusions to be equally applicable to DFT/MRCI.

While the generation of the reference space using selected CI is a core-feature and key strength of the DFT/MRCI(2) method, it also creates challenges in the construction of smooth PESs. In common with other selected CI methods, the general, nonfull CI configuration space generated via the selection procedure renders the corresponding adiabatic energies noninvariant with respect to orbital rotations, complicating the implementation of analytical gradients. More importantly, the reference space and first order interaction space generation procedure, as implemented in DFT/MRCI(2), results in PESs that are locally nonsmooth when the spaces generated are specific to each nuclear configurations. That is, it cannot be guaranteed that the DFT/MRCI(2) energies of a set of closely distanced structures constitute a reliably differentiable PES. However, the DFT/MRCI(2) PESs resemble continuous underlying surfaces that have been disrupted by deterministic noise that arises from the native selection procedure of the method, making it locally nonsmooth. As a result, implementations of standard numerical gradients are not guaranteed to yield physical or meaningful quantities and will be strongly dependent on the step size. A natural way to address the aforementioned problem is by building a smooth surrogate representation of the PES, on which standard numerical or analytical gradient schemes can be employed. To this end, Gaussian process regression (GPR) stands out for its simplicity and versatility. First, GPR performs well with small amounts of training data and is less data intensive compared to neural network architectures,^16^ thus making it suitable for on-the-fly generation of PESs. Second, the differentiability class of the predictor can be guaranteed by the choice of kernel, and different types of noise can be accounted for surprisingly well with a stochastic noise model in a straightforward manner.

Of particular importance in ultrafast excited state processes are regions of the PES where the upper and lower adiabatic energy surfaces cross and become degenerate. These points of degeneracy are called conical intersections (ConIns) and play an important role in ultrafast photochemical processes, such as radiationless decay. The construction of smooth quasi-diabatic potential surfaces at a conical intersection, employing only adiabatic energies has been previously explored, notably by Köppel and Schubert in the form of regularized diabatic states.^17^ Furthermore, the use of the column space of the squared-energy Hessian to characterize the branching space of two-state CoIns has been more recently further developed by a number of groups.^18−20^ The optimization of conical intersection structures without the need for explicitly constructing derivative coupling vectors has a similarly extensive history in the literature,^21^ including recent work that has explored the construction of smooth models in the vicinity of conical intersections.^22−25^

In this work, we will evaluate the ability of the DFT/MRCI(2) method to furnish GPR surrogate PESs that yield an accurate description of seams of conical intersection. This approach will be shown to obviate the need for the direct determination of gradients and derivative couplings on the nascent DFT/MRCI(2) surfaces. These surrogate-derived structures, as well as the corresponding branching (degeneracy-lifting) and seam (degeneracy-preserving) spaces, will be compared to the corresponding benchmark ab initio MRCI results. The demonstration that DFT/MRCI(2), when used to generate smoothly varying potential surrogates, can produce accurate seams of intersection is the first and necessary step toward excited state dynamics on GPR-derived PESs.

## 2 Computational Methods

### 2.1 Conical Intersection Optimization

We will here limit our discussion to the optimization and characterization of two-state ConIns. These are points in nuclear coordinate space where two adiabatic electronic states are degenerate, and where that degeneracy is lifted at first order in a two-dimensional coordinate subspace is termed the branching space. These points of degeneracy are not isolated, but form continuously connected seams of degeneracy in the remaining *N* – 2 coordinate subspace, where *N* is the number of internal degrees of freedom. One of the key approaches for characterizing this seam space is the optimization of minimum energy nuclear configurations on the seam. That is, minimum energy conical intersections (MECIs).

There are multiple approaches for optimizing MECIs,^26−29^ but most, including the techniques employed here, require the identification and utilization of the directions that span the two-dimensional branching space, which is defined by the energy difference gradient **g** and the derivative coupling vector **h**(30)^,^(31):
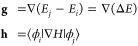
1where *E*_*j*_ and *E*_*i*_ are the upper and lower adiabatic energy surfaces, respectively, and |ϕ_*j*_⟩ and |ϕ_*i*_⟩ are the corresponding adiabatic states. *H* corresponds to the electronic Hamiltonian, and the gradient ∇ is taken with respect to the nuclear coordinates. The nascent branching vectors in eq 1 are in general not orthogonal, but can be orthogonalized by a rotation:
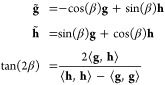
2where **g̃** and **h̃** are orthogonal and ⟨,⟩ refers to the inner product. The direct optimization^26^ approach, combined with the updated branching space method^21^ is employed for MECI optimizations here. The minimizing gradient **d** in the direct optimization method is given by
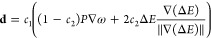
3where  is the average energy. We find that the parameters used in ref. Twenty-three worked well, namely *c*_1_ = 0.2 and *c*_2_ = 0.9, and in this work no Hessian is incorporated in the MECI optimization. *P* is the projector onto the seam space,
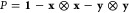
4where
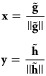
5correspond to the interaction adapted coordinates that span the branching space and thus are coindcident with the orthonormalized energy difference gradient and derivative coupling vector, respectively, and ⊗ refers to the outer product.

The adiabatic potential surfaces required to perform the MECI optimizations have discontinuous derivatives along the seams of degeneracy and are thus problematic to machine-learn. However, as detailed in refs (32), (33), all the required quantities can be reconstructed from the characteristic polynomial (CP) coefficients of the potential matrix. The fitting of PESs in regions of degeneracy via CP coefficients was first developed by Opalka and Domcke^33^ and has seen a renewal of interest.^34,35^

Let **V**(**R**) be the *n* × *n* adiabatic potential matrix for an *n*-state system, with on-diagonal elements *V*_*ii*_(**R**) corresponding to the adiabatic PESs *E*_*i*_(**R**). To separate out the parts that give rise to discontinuous derivatives at points of intersection, the potential matrix is decomposed into an average energy and splitting term:
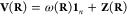
6where ω(**R**) = Tr[**V**(**R**)]/*n* corresponds to the average energy surface, and the splitting matrix **Z**(**R**) has elements *Z*_*ij*_(**R**) = [*E*_*i*_(**R**) – ω(**R**)]δ_*ij*_. The discontinuities in the derivatives of the adiabatic potentials manifest in the splitting elements *Z*_*ij*_(**R**), but a smooth representation can be obtained by considering its characteristic polynomial:
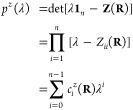
7with CP coefficients  given by
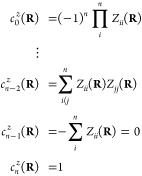
8The average energy surface ω(**R**) and the set of CP coefficient surfaces {} are smooth functions of the nuclear coordinates, even at points of ConIns, making them amenable to fitting. The adiabatic energies can subsequently be retrieved trivially as the eigenvalues of the Frobenius companion matrix
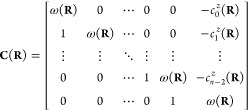
9For a two-state system, only the 0^th^ order CP coefficient  and the average energy ω(**R**) terms are present:
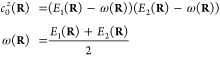
10with the corresponding Frobenius companion matrix:
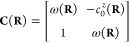
11the eigenvalues of which are the adiabatic energies, which have the following analytical form
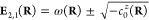
12

The optimizations are carried out directly on the ω(**R**) and  surfaces since they are smooth even at ConIns and amenable to learning.

### 2.2 Characterization of the Intersection Topography

**Figure 1:**
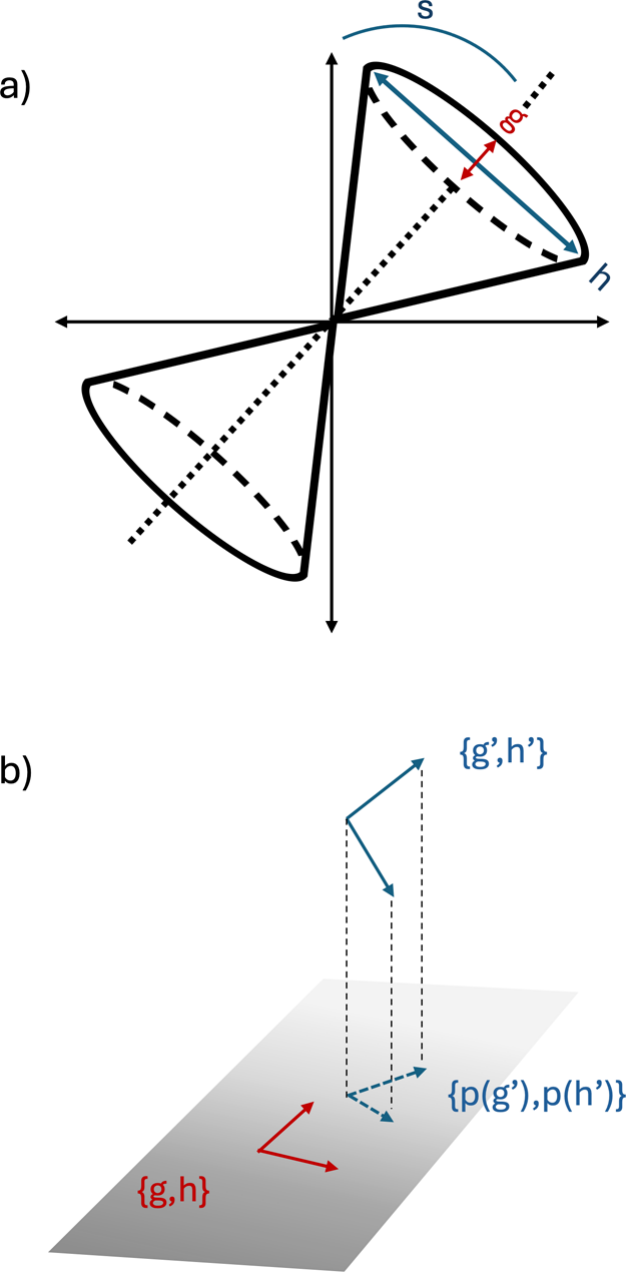
Illustration of the parameters shown in eqs 14 and 17. The parameter *s* shows the tilt of the double cone’s principle axis, and *g* and *h* are the norms of the energy difference gradient and derivative coupling vector, respectively, and describes the asymmetry or ellipticity of the cone in the first order picture.

To characterize the conical intersection topography at the optimized MECI geometry, we first determine the intersection adapted **x** and **y** (eq 5) coordinates that span the branching space. When the derivative coupling vector is not available, the branching space vectors can also be obtained from the curvature information on the energy difference squared surface Δ*E*^2^(**R**). Specifically, for a two-state CI, the eigenvectors associated with the nonzero eigenvalues of the energy difference squared Hessian form the branching space.^18,32^ In the ω–CP model, this Hessian is equivalent to taking the Hessian of .^32^ This was the approach employed here to determine the branching space vectors at MECIs optimized on DFT/MRCI(2) derived GPR-surrogates. The intersecting potentials are described by a first-order two-state model given by^1^
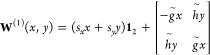
13where *g̃* and *h̃* are the norms of the orthogonalized energy difference gradient and derivative coupling vector, respectively. The magnitudes of the *s*_*x*_ and *s*_*y*_ parameters characterize the tilt of the cone relative to the branching plane normal vector,
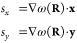
14These four parameters (*g̃*, *h̃*, *s*_*x*_, *s*_*y*_) are employed to characterize the topography of the double cone to first order. However, these parameters alone do not provide an obvious connection to the coupled electronic and nuclear dynamics that may result via passage through this region of the PES. Recent work by Lindh and co-workers^36^ resulted in the definition of composite parameters that correlate to possible reactive outcomes following passage through the region of the CI. The authors defined the following two parameters:
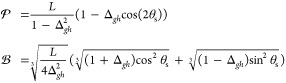
15where the intersection asymmetry, Δ_*gh*_, and the parameters *L* and θ_s_ are given by
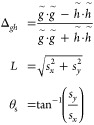
16For  1, the CI corresponds to a peaked (sloped) intersection, and for  1, the CI has a bifurcating (single-path) intersection. In short, a CI is peaked (sloped) if the upper surface is (is not) at a minimum in the branching plane, and it is single-path (bifurcating) if there is only one minimum (multiple minima) on the lower surface in the branching plane. Another metric to directly compare two branching spaces is by projecting the branching space vectors of one method onto the branching plane of the other.^36,37^ More precisely, let {**x**, **y**} and {**x***′*, **y***′*} be the sets of orthonormalized branching space vectors for method *I* and *J*, respectively, then the area enclosed by the unit branching vectors of method *I* projected onto the branching plane of method *J* is given by
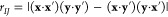
17The value *r*_*IJ*_ is bounded between 0 for orthogonal branching planes and 1 for parallel branching planes. The parameters introduced above are illustrated as cartoons in Figure 1. Together, the MECI can be characterized with the parameters *g̃*, *h̃*, *s*_*x*_, *s*_*y*_,  and  defined in eqs 14 and 15, and the branching spaces of different methods can be compared with *r*_*IJ*_ defined in eq 17, which we use to compare the optimized MECIs of DFT/MRCI(2) to those of ab initio MRCI.

### 2.3 Gaussian Process Regression Surrogate Construction

The training nuclear geometries were generated using atom-centered Cartesian Latin Hypercube Sampling (LHS), and the Smooth Overlap of Atomic Positions (SOAP) descriptor was used. We found a default (see Table S7) set of SOAP parameters that worked well and these were used for all calculations. Since this work focuses on the GPR surrogate representation of DFT/MRCI(2) seams of intersection and the subsequent characterization of MECIs, we start from geometries close to the MECI and build a static surface on which a CI optimization is performed. We thus remove the requirement for active learning; namely the optimal approach for updating the surrogate and hyperparameters during the course of the MECI optimization. The employment of an active learning process will be important for the practical deployment of the approach for the optimization of structural minima, but would add additional complexity to the present proof-of-principles study and is instead reserved for future work. More Information on the SOAP parameters and initial geometries are included in the SI.

We have chosen to approximate the local discontinuous variations of the DFT/MRCI(2) potentials as independent and identically distributed (i.i.d.) Gaussian noise within the GPR framework, which has a simple and closed form method of determination via the optimization of a whitenoise kernel. It is important at this point to reinforce the point that DFT/MRCI(2) PESs exhibit deterministic noise, but treating it as a i.i.d. Gaussian noise within the GPR framework leads to significant formal simplifications. Local energetic variation herein refers to the slightly different reference spaces generated at different geometries that renders DFT/MRCI(2) potential energy surfaces locally nonsmooth. We use a simple isotropic radial basis function (RBF) combined whitenoise kernel to model i.i.d. homoscedastic noise.
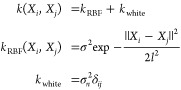
18where σ^2^, *l*^2^, and  are the kernel hyperparameters that control the variance, length scale and noise, respectively. *X*_*i*_ and *X*_*j*_ are the inputs of the function being learned, in this case the SOAP power spectrum corresponding to different nuclear geometries. The kernel gives the pairwise correlation between different inputs.

Given a kernel, the variant of GPR employed in this work then returns a predictor
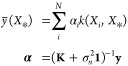
19where **y** is a vector of the training values, in this case the ω and  values, X_*_ is a point of prediction, and **K** is the *N* × *N* Gram matrix for *N* training points:
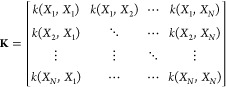
20

Herein, the learned GPR predictor of a function *y*(*x*) is denoted with an overbar, as *y̅*(*x*). The set of kernel hyperparameters is  are determined by maximum likelihood estimation (MLE).^38^ This differs from the majority of works done on the GPR learning of energy surfaces,^39−43^ which does not explicitly account for noise inherent to the method but rather employs a regularization term. Although a simple difference, it has strong consequences on the MECI optimizations as we will discuss subsequently. An important point of note here is, although we are modeling and fitting the local energetic variation as i.i.d. Gaussian noise, they are in fact more appropriately considered as deterministic noise. For example, multiple random samples of the energy at the same structure yields a delta distribution, and averaging over a large sample does not cancel out the noise. Regardless, this model provides a convenient formalism for handling the local energetic variations and learning a smooth surface. An additional assumption made here is that the local energetic variations within a set of closely distanced structures, , are small compared to the total energetic variations across the surface of interest, , so that .

For the DFT/MRCI(2) MECI optimizations, we consider a distribution of the parameters *g̃*, *h̃*, *s*_*x*_, *s*_*y*_, ,  and *r*_*IJ*_ computing a set of 50 surrogates, each constructed with different LHS centered at the same geometry. The reason for this is that we found that using atom-centered Cartesian LHS to generate the training data can result in deviations between different learned surrogate surfaces. This can then manifest itself in slightly different optimized MECI geometries and branching space topographies. This dependence on the training data is expected due to GPR being a nonparametric method. In contrast to parametric fitting, GPR does not assume a functional form, but is based solely on the correlation between each training point, given by the choice of kernel, thus its sensitivity to the training set. We have found this to be a more general problem, and contend that nuclear configurations generated from LHS will not be ideally suited to the on-the-fly construction of consistent, high-dimensional PESs when the kernel hyperparameters are obtained from MLE. In addition to the curse of dimensionality from which LHS suffers,^44^ it has been shown that GPR hyperparameters optimized with MLE from data generated with LHS can perform poorly due to irregular pairwise distance distribution.^45,46^ The question of optimal sampling and fitting, however, is not addressed in this work and we stick to atom-centered Cartesian LHS. To confirm the observed sensitivity does not instead originate from the underlying DFT/MRCI(2) calculations, however, the same procedure of constructing multiple different surrogates was employed using ab initio MRCI energies, and the same sensitivity of the parameters on the training data was found. This will be discussed in more detail subsequently.

### 2.4 Electronic Structure Methods

The primary objective of this work is to optimize MECI structures on surrogate surfaces derived from DFT/MRCI(2) potential energy surfaces. To this end, the DFT/MRCI(2) method was employed to compute single point electronic energies in the vicinity of seams of conical intersection. Previous work has shown the (approximate) DFT/MRCI(2) approach to furnish excitation energies in near-quantitative agreement with the parent DFT/MRCI method.^15,47^ The density fitted^48,49^ cc-pVDZ basis set was used in all cases as implemented in the PySCF package, and 3 roots were computed for all the molecules studied. The initial, guess reference space was generated using the previously discussed AutoRAS procedure.^50^ The General Reference Configuration Interaction (GRaCI) package was used for all DFT/MRCI(2) computations,^51^ where the DFT portion of the calculation was computed using PySCF.^52−54^

For comparison, the MECIs and corresponding branching spaces were also determined using ab initio MRCI methods. MR-CI singles (MR-CIS) and MR-CI singles and doubles (MR-CISD) MECIs were optimized, employing state-averaged complete active space self-consistent field (SA-CASSCF) reference wave functions, where *m*-electron, *n*-orbital active spaces, and number of roots in the averaging procedure, *S*, are specified *S*-(*m*, *n*) and vary by molecule. For ethylene, butadiene, and fulvene, these correspond to 2-(2,2), 2-(4,4), and 2-(6,6), respectively. The orbitals included in the active space for each of the molecules are included in the Supporting Information (SI). The COLUMBUS electronic structure package was used for all ab initio MRCI computations.^55^ All optimized structures are provided as Supporting Information in zip files.

## 3 Results

In the following, we present MECIs obtained via optimization on surrogate potentials that are determined from DFT/MRCI(2) single point energies, and compare it to the MECIs computed using analytical ab initio MRCI gradients and nonadiabatic couplings. The representative examples discussed below have been previously studied in detail: the accidental *S*_0_/*S*_1_ ConIns of “twisted-pyramidalized” ethylene,^56−58^ “transoid” butadiene,^59,60^ and “twisted” fulvene,.^61^

**Figure 2:**
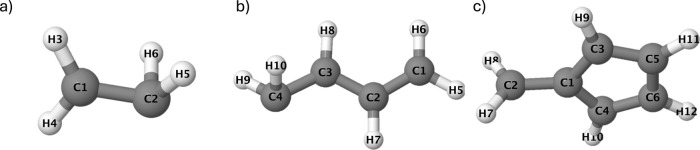
MECI geometries optimized using GPR surrogates of DFT/MRCI(2) potentials for (a) ethylene, (b) butadience and (c) fulvene. The atom numbers are also labeled, which is used to define the internal coordinates of interest.

The Cartesian geometries of all the optimized MECIs from the different sampled surrogates are provided as Supporting Information. However, a comparison of key internal coordinates between the mean DFT/MRCI(2) structures, computed from the optimized MECI over the 50 surrogates constructed from different training data, and those obtained using ab initio MRCI is shown in Table 1. The nuclear configurations for each of the structures discussed below are shown in Figure 2, where the atom numbering employed will be used throughout the subsequent discussion. The overlap of each individual DFT/MRCI(2) surrogate-derived MECI geometry with the corresponding MR-CISD structure are shown in Figure S2 of the SI.

**Table 1:** Comparison of Optimized Internal Coordinates for the *S*_0_/*S*_1_ MECI of Ethylene, Butadiene and Fulvene^a^

		*E*_MECI_	*r*_C_1_C_2__	θ_H_4_C_1_H_3__	θ_H_5_C_2_H_6__	γ_C_1_H_5_H_6_C_2__	
Ethylene	DFT/MRCI(2)	4.85 ± 0.01	1.43 ± 0.01	114.2 ± 1.424	93.70 ± 1.08	62.76 ± 1.124	
	MR-CIS	5.51	1.42	113.7	93.00	59.97	
	MR-CISD	4.99	1.41	112.4	94.3	61.39	

		*E*_MECI_	*r*_C_1_C_2__	*r*_C_2_C_3__	*r*_C_3_C_4__	θ_H_5_C_1_H_6__	γ_C_3_H_9_H_10_C_4__
Butadiene	DFT/MRCI(2)	4.34 ± 0.02	1.37 ± 0.002	1.40 ± 0.002	1.44 ± 0.004	117.63 ± 0.19	62.77 ± 0.26
	MR-CIS	4.30	1.33	1.45	1.43	116.84	65.00
	MR-CISD	4.24	1.35	1.45	1.42	117.11	65.74

		*E*_MECI_	*r*_C_1_C_2__	*r*_C_1_C_3__	*r*_C_3_C_5__	θ_H_7_C_2_H_8__	τ_H_7_C_2_C_1_C_3__
Fulvene	DFT/MRCI(2)	2.91 ± 0.06	1.45 ± 0.009	1.41 ± 0.007	1.41 ± 0.004	116.5 ± 1.269	89.99 ± 0.007
	MR-CIS	2.71	1.48	1.42	1.43	118.2	90.00
	MR-CISD	2.61	1.48	1.43	1.43	118.05	90.00

a The energy of the MECI is given in eV relative to the ground state minimum. The coordinate *r*_*xy*_ is the distance between atoms *xy* and is given in Å. The angles θ_*xyz*_ (angle between atoms *xyz*), γ_*wxyz*_ (out of plane angle of atom *w*), and τ_*wxyz*_ (dihedral angle of atoms *wxyz*) are given in degrees.

The DFT/MRCI(2) optimized MECI parameters for ethylene, butadiene and fulvene are summarized in Tables 2 and 3. Table 2 compares the DFT/MRCI(2) MECI topographies to those obtained from ab initio MRCI methods, while Table 3 shows the overlap of the branching spaces for each of the methods. For rows and columns corresponding to DFT/MRCI(2), the presented value is the arithmetic mean ± the standard deviation over the values obtained from the set of 50 surrogates.

**Table 2:** Comparison of DFT/MRCI(2), MR-CIS and MR-CISD MECI Topographies^a^

		*g̃*	*h̃*	*s*_*x*_	*s*_*y*_		
Ethylene	DFT/MRCI(2)	0.209 ± 0.002	0.181 ± 0.004	0.056 ± 0.011	0.019 ± 0.005	0.087 ± 0.026	1.526 ± 0.198
	MR-CIS	0.213	0.125	0.130	0.046	0.507	1.289
	MR-CISD	0.204	0.129	0.102	0.029	0.303	1.174
Butadiene	DFT/MRCI(2)	0.224 ± 0.002	0.129 ± 0.001	0.032 ± 0.007	0.032 ± 0.001	0.082 ± 0.015	0.602 ± 0.062
	MR-CIS	0.207	0.110	0.133	0.023	0.452	1.114
	MR-CISD	0.183	0.109	0.123	0.023	0.492	1.268
Fulvene	DFT/MRCI(2)	0.187 ± 0.056	0.172 ± 0.005	0.036 ± 0.032	4.90 × 10^–4^ ± 3.2 × 10^–3^	0.052 ± 0.116	1.902 ± 0.742
	MR-CIS	0.187	0.180	0.047	5.89 × 10^–5^	0.063	2.414
	MR-CISD	0.202	0.199	0.032	1.74 × 10^–3^	0.025	3.637

a For columns and rows corresponding to DFT/MRCI(2), the value presented is the arithmetic mean ± the standard deviation over values obtained from 50 surrogates.

**Table 3:** Comparison of DFT/MRCI(2), MR-CIS and MR-CISD Branching Spaces^a^

	*r*_*IJ*_	DFT/MRCI(2)	MR-CIS	MR-CISD
Ethylene	DFT/MRCI(2)	1.000	0.950	0.988
	MR-CIS	0.950	1.000	0.978
	MR-CISD	0.988	0.978	1.000
Butadiene	DFT/MRCI(2)	1.000	0.975	0.962
	MR-CIS	0.975	1.000	0.981
	MR-CISD	0.962	0.981	1.000
Fulvene	DFT/MRCI(2)	1.000	0.975	0.976
	MR-CIS	0.975	1.000	0.999
	MR-CISD	0.976	0.999	1.000

a Values are bounded between 0 and 1 for parallel and orthogonal branching planes, respectively.

Turning first to the comparison of the structural parameters, we find that the DFT/MRCI(2) MECIs are generally in good agreement with the structures optimized using ab initio MRCI methods, as shown by the similarity in the internal coordinates. Deviations in bond lengths and bond angles are typically within 0.05 Å and 3°, respectively. Since they are not the same level of theory, this deviation is well within reasonable agreement. Furthermore, the corresponding energy of the DFT/MRCI(2) surrogate-optimized structures, given in Table 1 relative to the ground state minimum, are in excellent agreement with the ab initio MR-CIS and MR-CISD results. The relative energies of the DFT/MRCI(2)-derived structures exhibit differences of <0.3 eV compared to MR-CISD for each of the molecules studied.

The good agreement between the DFT/MRCI(2) and ab initio MRCI structural parameters is further mirrored in the comparison of the branching space parameters summarized in Table 2, but with some caveats. The norms of the branching space directions, *g̃* and *h̃*, are in good agreement between all methods and levels of theory except for ethylene, which exhibits a slight difference in the norm *h̃*. The deviation, however, does not give rise to a qualitatively different ConIn region, as both methods furnish an asymmetric double cone. However, there appears to be a consistent difference between the DFT/MRCI(2) and MRCI tilt parameters *s*_*x*_ and *s*_*y*_. Specifically, these parameters are consistently smaller for the DFT/MRCI(2) branching spaces in comparison to the ab initio MRCI results for the examples considered here, resulting in former furnishing intersections characterized by vertical nonsloped cones.

While the absolute magnitude of the tilt parameters may exhibit large differences, we see that if the ratio of *s*_*x*_/*s*_*y*_ is similar, then the composite parameters  and , defined in eq 15, may be similar. For example, although the DFT/MRCI(2) tilt parameters for ethylene are significantly smaller than their ab initio MRCI counterparts, the predicted  and  values fall under the same category. That is, they both predict peaked and single-path intersections. On the other hand, for butadiene, not only are the DFT/MRCI(2) tilt parameters a lot smaller, the ratio is also significantly different. As a result, DFT/MRCI(2) predicts a bifurcating intersection, whereas MRCI predicts a single-path intersection,

These differences in the intersection topography discussed above do not translate, however, into significant differences in the respective branching spaces. As shown in Table 3, the DFT/MRCI(2) and ab initio MRCI branching planes are all nearly parallel, which manifests in near-unit *r*_*IJ*_ values.

**Figure 3:**
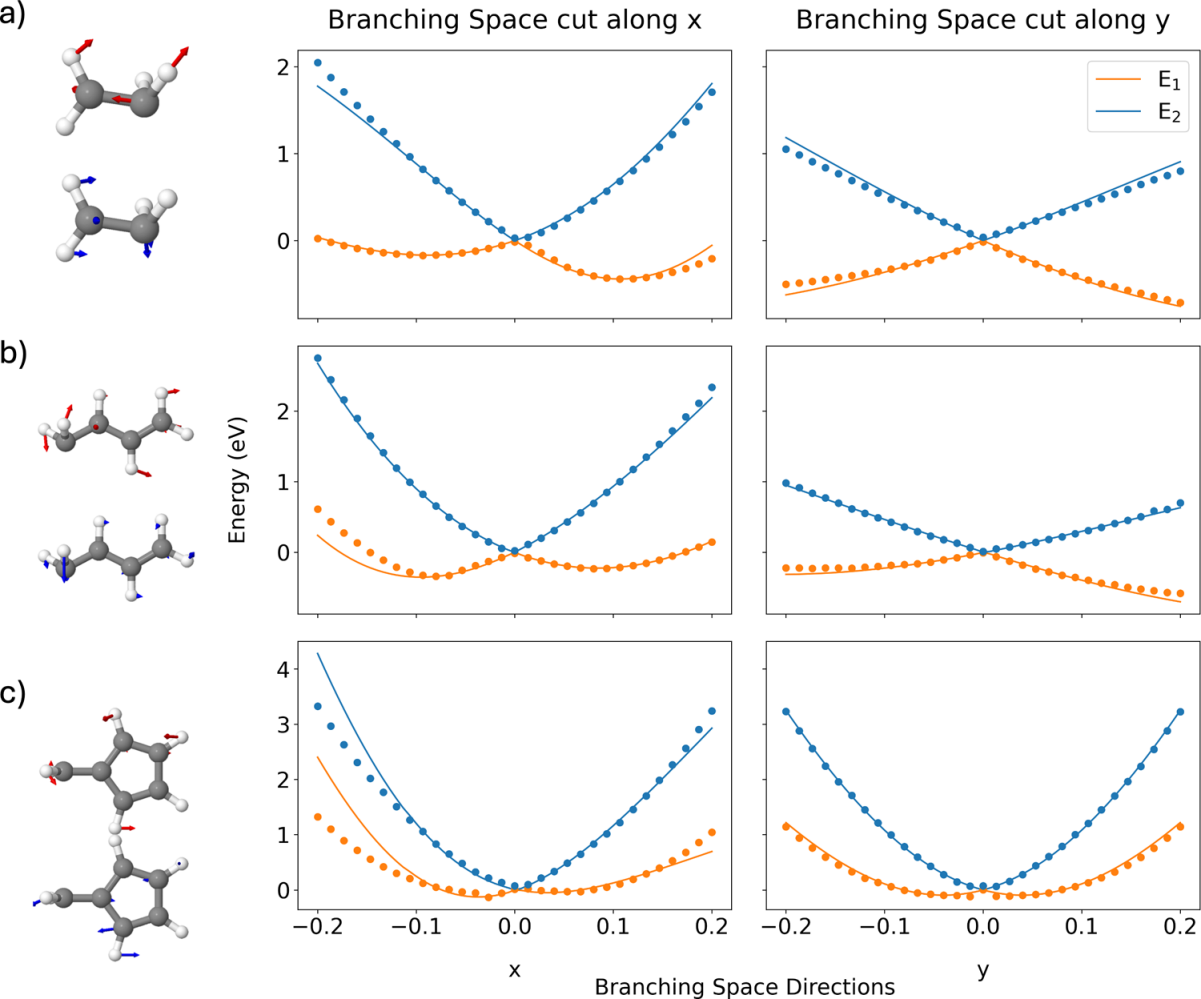
Adiabatic energies in the vicinity of the MECI along the branching vectors for: (a) Ethylene, (b) Butadiene, (c) fulvene. Left panel shows the optimized MECIs and their corresponding **x** (red) and **y** (blue) branching vectors. The middle and right panel shows the Adiabatic energies along the **x** and **y** branching vectors, where the dotted points correspond to the DFT/MRCI(2) energies and the solid lines correspond to the GPR predicted energies.

Finally, to confirm that the surrogate potentials accurately reproduce the DFT/MRCI(2) potential surfaces within the branching space, Figure 3 shows DFT/MRCI(2) single point energies computed along the branching space directions, with the origin corresponding to the MECI geometry, for ethylene, butadiene and fulvene. Figure 3 shows that the GPR surrogates reproduce well the DFT/MRCI(2) energies in the vicinity of the MECI along both branching space directions. To our knowledge, these determinations correspond to the first explicitly computed DFT/MRCI ConIn branching spaces.

**Figure 4:**
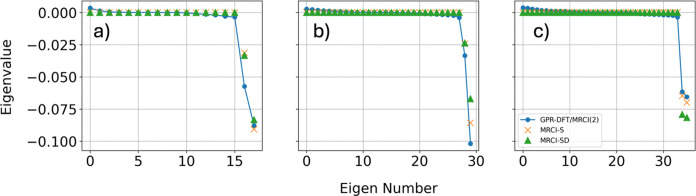
Eigenvalues of the  Hessian for: (a) Ethylene, (b) Butadiene, (c) Fulvene. The dotted values are the GPR predicted values for DFT/MRCI(2), and the cross and triangle points are the ab initio MRCI-S and MRCI-SD values, respectively. The number of nonzero (zero) elements correspond to the branching (seam) space dimension.

Additionally, Figure 4 shows the eigenvalues of the  Hessian, with the number of nonzero (zero) eigenvalues corresponding to the branching (seam) space dimension. The first point of note is that the eigenvalues of the seam space coordinates should be strictly zero. For DFT/MRCI(2), the seam space eigenvalues show small deviations from zero, but is within the uncertainty of the GPR surrogate model, which will be discussed in Section 4.3. In practice, Figure 4 shows that DFT/MRCI(2) is able to correctly capture the branching and seam space dimensions.

## 4 Discussion

### 4.1 Evaluation of DFT/MRCI(2) MECI Regions

As shown above, the DFT/MRCI(2)-derived potential energy surfaces furnish seams of intersection, including MECIs, that are, in general, similar to ab initio MRCI. Comparison of the key internal coordinates for the MECIs as well as the overlap of the respective branching spaces, show that the level of agreement between all pairs of methods is similar. Aside from the small difference in *h̃* for ethylene, the remaining branching space parameters norms are well produced by DFT/MRCI(2). From these observations, we can conclude that the branching spaces of DFT/MRCI(2) and ab initio MRCI are essentially the same for the examples considered here. That is, DFT/MRCI(2) near-quantitatively reproduces the branching spaces of MR-CISD for each of the paradigmatic ConIns studied. One notable exception to this agreement are the tilt parameters. The GPR-determined values for *s*_*x*_ and *s*_*y*_ indicate that DFT/MRCI(2) surfaces yield vertical nonsloped cones in contrast to the ab initio MRCI results, which predict sloped intersections. A more detailed analysis of this observation will be presented in Section 4.2.

Even given this difference, we would like to note that the level of agreement with MR-CISD is still very good. For comparison, a previous study by Filatov and co-workers and Lindh and co-workers compared the branching spaces resulting from different electronic structure methods to MR-CISD for a range of molecules.^36,37^ The methods examined included SA-CASSCF, ensemble DFT approaches,^62,63^ and the orthogonalization-corrected (OM2) semiempirical configuration interaction method.^64,65^ As that study demonstrated, the branching spaces furnished by different electronic structure approaches can vary widely, with, for example, spin-flip TDDFT (SF-TDDFT) yielding *r*_*IJ*_ overlaps with the benchmark MRCI ranging from 0.64 for a twisted-bond-alternating methylimine MECI to near quantitative agreement and a 0.986 overlap for methylimine MECI. Thus, the average overlap between the DFT/MRCI(2) and MR-CISD branching spaces of 0.98 for the examples considered should be considered near quantitative agreement.

Furthermore, while MECI structures predicted from the ab initio MRCI methods and DFT/MRCI(2) are in very good agreement, the corresponding intersection topographies exhibit some minor differences arising from the absolute magnitude of the tilt parameters. However, the ratio of these quantities *did not* exhibit systematic differences and thus comparisons of metrics intended to be predictive of reactive outcomes ( and  of eq 15) following passage through a ConIn were in general favorable.

Summarizing these observations, we find that the optimized MECI structures, as well as the branching spaces of DFT/MRCI(2) and ab initio MRCI are in near-quantitative agreement. The CI topography, however, exhibits systematic differences from ab initio MRCI approaches in the form of smaller DFT/MRCI(2) tilt parameters *s*_*x*_ and *s*_*y*_.

### 4.2 Comparison of DFT/MRCI(2) and Ab Initio MRCI Surrogate Surfaces

As shown above, the primary difference between the DFT/MRCI(2) and ab initio MRCI MECI topographies is the tilt of the double cones. Here, we investigate this finding in more detail and confirm that these deviations represent real differences in how the electronic structure methods describe the seam of intersection, and are not artifacts of the surrogate generation procedure.

To this end, GPR surrogates for the twisted-pyramidalized MECI of ethylene were constructed using MR-CIS single point energies. To compare the ω̃ and  GPR surrogates computed using MR-CIS and DFT/MRCI(2) energies, we consider the resulting learned kernel hyperparameters. These values are given in Table 4. The difference in the tilt parameters can then be directly related to the learned hyperparameters. It can be seen that apart from the noise term, the learned variance σ^2^ and length scale *l* of  is similar between DFT/MRCI(2) and MR-CIS. The straightforward interpretation of these results is that the two learned surfaces are similar in terms of how large and how rapidly the  surface is varying around a MECI, which are reflected in the length scale and variance, respectively. The DFT/MRCI(2)-based surrogates have a larger noise term to account for the local energetic variations. The two ω̅(**R**) surfaces, however, are different, with the DFT/MRCI(2)-based surrogate having appreciably smaller σ^2^ and *l* hyperparameters. Examining eq 14 and taking the gradient of ω̅(**R**), we can see that the tilt parameters are proportional to σ^2^ and inversely proportional to *l*,
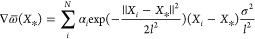
21

**Table 4:** MLE Optimized GPR Hyperparameters of Ethylene for DFT/MRCI(2) and MR-CIS^a^

		DFT/MRCI(2)	MR-CIS
	σ^2^	316^2^ ± 0.0	316^2^ ± 0.0
	*l*	0.095 ± 0.002	0.111 ± 0.001
		0.011 ± 0.001	2.52 × 10^–6^ ± 5.36 × 10^–7^
ω	σ^2^	40^2^ ± 20^2^	277^2^ ± 98^2^
	*l*	0.088 ± 0.007	0.323 ± 0.010
		0.0023 ± 0.0001	3.27 × 10^–8^ ± 4.54 × 10^–9^

a The first and second row corresponds to the learned hyperparameters for the  and ω̅(**R**) surface, respectively. The values presented are the arithmetic mean ± standard deviation over 50 surrogates.

Additionally, from the computed **g̃** and **h̃** norms as well as the large overlap between the DFT/MRCI(2) and MR-CIS branching spaces, we can exclude differences in the branching vectors as the reason for the difference in the tilt parameters. That is, the norms of **x** and **y** in eq 14 is not the dominant term that results in smaller values of *s*_*x*_ and *s*_*y*_ for DFT/MRCI(2) compared to MR-CIS. The significantly smaller value of σ^2^ for DFT/MRCI(2) is thus found to be responsible for giving rise to smaller values of *s*_*x*_ and *s*_*y*_.

There is a simple rationalization of the difference between the DFT/MRCI(2) and MR-CIS  surfaces, which differ only in the noise term. Namely, that the MR-CIS potential surfaces do not suffer from the presence of deterministic noise that is inherent to the DFT/MRCI(2) method. However, the same conclusion cannot be drawn for the differences in ω̅(**R**). Since we cannot provide physically motivated reasons for the difference in the log-likelihood landscape that arise due to different electronic structure methods, the MLE optimized noise term does not offer insight into the physics of the DFT/MRCI(2) local energetic variation. When the set of optimized hyperparameters  differ significantly, they may correspond to extrema of drastically different log-likelihood landscapes, thus frustrating a simple interpretation. Furthermore, it is also important to remember that the whitenoise kernel hyperparameter σ_*n*_^2^ is not an absolute measure of noise. Depending on the kernel composition, the log-likelihood surface can differ, even employing the same training data, thus using different kernels changes the learned σ_*n*_^2^ value significantly. More importantly, different metric spaces or cost functions used in the training can result in drastically different sets of optimized hyperparameters, and subsequently change the role and interpretation of the noise term in the model. For the analysis performed here, we assume that the PESs in the vicinity of the MECI computed using DFT/MRCI(2) and ab initio MR-CIS do not have significant qualitative differences, which would otherwise make the use of the same kernel inappropriate. Therefore, although the i.i.d. noise model provides a simple and intuitive picture, it should only be interpreted as a smoothing device in the construction of PESs. The main consequence from all the above is, the DFT/MRCI(2) ω(**R**) surface is responsible for the smaller tilt parameters, but we cannot conclude from the hyperparameters alone that the DFT/MRCI(2) and MR-CIS ω(**R**) surfaces are similar to added deterministic noise for DFT/MRCI(2), as in the case of the  surfaces, because the optimized hyperparameters are drastically different.

### 4.3 Fundamental Uncertainty in DFT/MRCI(2) Derived GPR-Surrogates

Before turning to the main challenges that arise from the present framework, we first address the uncertainty corresponding to the surrogate dependence on different LHS distributions. To study the sampling dependence of the GPR-surrogates, 50 surrogates were also constructed at the MR-CIS level of theory by LHS about the MECI geometries. MECI optimization was performed on each of the 50 GPR surrogate surfaces, and the mean and standard deviation of the parameters reported in Table 2 were also determined for this data set. For each MECI optimization, the geometry with the largest Δ*E* in the training set was chosen as the starting geometry in order to keep the initial conditions consistent. As expected, the surrogate dependence on LHS is also present in the MR-CIS derived surfaces, and the standard deviations on the branching space parameters is of the same order of magnitude. These results are summarized in Table S3.

These results demonstrate that there is a non-negligible sampling dependence to the resulting surrogates, regardless of the electronic structure method employed to generate the electronic energies. This can also be seen more directly from Table 4, which shows the distribution of learned hyperparameters over the set of 50 surrogates. This error is clearly always present when fitting surfaces using training data generated from LHS distributions, and although usually small, it is particularly relevant when points of degeneracy are sought. It is not clear how the dependence of the determined surrogate potentials on the nuclear geometry sampling scheme may be significantly reduced or eliminated, and a more detailed examination of this issue is reserved for a future work.

Furthermore, while this sampling dependence is present in both the MR-CIS and DFT/MRCI(2) derived surrogates, a major difference is the value of the learned noise hyperparameter : it is significantly larger for DFT/MRCI(2) compared to MR-CIS. Note that since these hyperparameters are learned from data transformed to have zero mean and unit variance, and as a result it does not correspond to a physically interpretable value. Nevertheless, by comparing the learned DFT/MRCI(2) and MR-CIS  values, shown in Table 4, it is clear that the learned DFT/MRCI(2) ω(**R**) and  surface is a lot noisier compared to MR-CIS due to the local energetic variations. This is not unexpected, since each nuclear configuration results in the generation of a new reference space, but also highlights the need for surrogate representations of the nascent DFT/MRCI(2) surfaces in order for them to be meaningfully differentiable.

**Figure 5:**
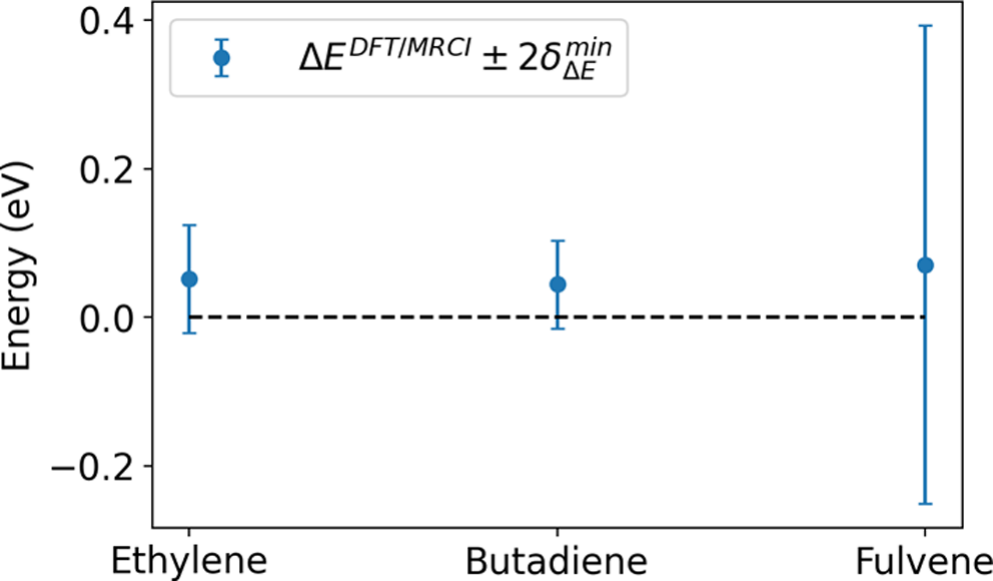
Effect of noise as a learned hyperparameter on the achievable accuracy of the optimized DFT/MRCI(2) MECI degeneracy. The blue dots shows the DFT/MRCI(2) energies at the optimized MECI, and the error bar shows the 95% confidence interval as a result of the learned noise. The true degeneracy (dashed line) is contained in the 95% confidence interval for all the molecules.

An important consequence of the larger noise in the DFT/MRCI(2) potentials is that it translates into a limitation on the level of degeneracy that can be achieved. The GPR predictor returns a mean *y̅*(X_*_) and standard deviation δ_*y̅*_(X_*_) by treating the target *y* as a Gaussian random variable. Within the GPR framework, we can then say that the real value *y*(X_*_) falls between the interval *y̅*(X_*_) ± 2δ_*y̅*_(X_*_) with a 95% confidence. For a noiseless GPR model, ignoring the presence of a small regularization term, the predicted variance of a training point is zero:
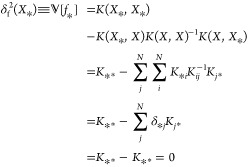
22where we have made use of the fact that *K* is symmetric and that for , where  is the training set, the sum over *i* on the second line becomes a Kronecker-δ function. In a noiseless model, adding a training point collapses the predicted variance (and standard deviation) at that point to zero. Thus, a model can be made arbitrarily accurate within a region by adding training points, provided the covariance matrix does not become ill-conditioned. The lower bound on the predicted standard deviation for a noiseless model is therefore zero, such that  for , where  corresponds to a minimum uncertainty given in terms of standard deviation. In our model, however, a nonzero lower bound on the predicted variance exists at any point due to the learned noise term, even if the point is in the training set. That is, eq 22 does not hold in the presence of noise because the sum over *i* does not become a Kronecker-δ function when the inverse takes on the form of . As a result,  for . The presence of noise thus renders the minimum uncertainty nonzero, which means that the degeneracy can only be meaningfully determined up to the lower bound before adding additional points becomes irrelevant. Note that this statement holds on the condition that reoptimizing the GP hyperparameters with additional training points does not decrease the learned , which we found to be the case for the systems under study. Since the minimum uncertainty is learned in terms of , it is propagated forward to Δ*E*(**R**) in the small value limit as^66^
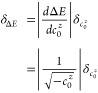
23recalling that . The result of this is illustrated in Figure 5, where the DFT/MRCI(2) Δ*E* at the MECI is nonzero, and the degeneracy falls within the 95% confidence interval of the minimum uncertainty. That is,
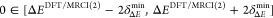
24For comparison, the MR-CIS minimum standard deviation  for ethylene was found to be 6 × 10^–4^ eV, which is consistent with the level of convergence employed in the calculation of the electronic energies. A major shift in interpretation resulting from treating the local energetic variations in DFT/MRCI(2) as noise is the role of the single point calculations in the construction of surrogate PESs. It is no longer taken as the ground truth and is only meaningful within an interval governed by the learned noise. A consequence of this is that the degeneracy may not exist simultaneously in the DFT/MRCI(2) PESs and the GPR surrogate, and if it does, it may not correspond to the same set of geometries unless the noise approaches zero.

At this point it is important to discuss a challenge that arises when learning the  surface. In the direct MECI optimization approach used here, the minimizing gradient is separated into two components. The first minimizes the average energy projected onto the seam space, and the second component minimizes a quantity proportional to Δ*E*^2^, with its minimizing gradient as 2Δ*E*(∇ **x̂**), where **x̂** is the normalized energy difference gradient. For the ω – CP model,  is a strictly negative and real quantity for any **R**. Problems will arise whenever the solution gets near the degeneracy and the  surrogate returns a positive, albeit small value, which renders the predicted Δ*E*, and subsequently the gradient component responsible for minimizing the degeneracy, to be zero, since we only take the real parts of the gradient. Thus, the degeneracy cannot be made arbitrarily small with increased iterations. To ensure that the branching space cuts in the vicinity of the origin do not exhibit spurious cusps due to positive  values, all the  values were shifted with  in the branching space cut plots, which also forces the origin and Δ*E̅*(**R**_MECI_) to be zero.

These two issues described in this section are the main challenges of DFT/MRCI(2) MECI optimization via GPR surrogates. The first is quantifiable by examining the minimum uncertainty of the degeneracy , and is to be expected since we are essentially smoothing a noisy surface. The second challenge, maintaining a physical sign on the  surface, is related to the machine learning architecture employed. Whether the physical constraints of a positive  surface can be learned given enough data or requires a built-in constraint is an open question. There has been recent work addressing this issue, including designing neural network architectures^34^ and employing different companion matrices.^35^ In the case of GPR, this issue is intimately related to the Gaussian noise model used, where the noise is symmetrical about the mean with infinite support and cannot guarantee a nonpositive value. This issue can be naively remedied in a noiseless setting by adding training points where nonphysical  is predicted, or more generally and robustly, even in the noised setting, by considering bounded GPR,^67^ which includes the use of warped-GPR, non-Gaussian likelihood or constrained kernel hyperparameter optimization, and may be worth exploring in future work.

## 5 Conclusions

In this work, we have evaluated the ability of DFT/MRCI(2) to describe seams of conical intersections using a GPR surrogate approach. To obviate the need to learn adiabatic surfaces, which exhibit cusps at points of degeneracy, we instead learned the coordinate dependent coefficients of the characteristic polynomial of the potential matrix, which are smooth functions of nuclear coordinates. We found that the explicit inclusion and optimization of a whitenoise kernel allows for an intuitive framework to learn smooth surrogate DFT/MRCI(2) surfaces that are otherwise discontinuous due to the selected CI aspects of the method. Using the GPR surrogate characteristic polynomial coefficients, we optimized the MECIs of ethylene, butadiene and fulvene and computed for the first time the corresponding branching space and topography using DFT/MRCI(2). The asymmetry of the double cones at the optimized MECIs agree well with ab initio MRCI, but the tilt parameters are smaller in magnitude, indicating that DFT/MRCI(2) preferentially gives vertical double-cones compared to ab initio MRCI for each of the molecules studied. The difference in the optimized hyperparameters of the average energy surrogates, combined with the good agreement between the branching spaces, shows that the difference in the tilt parameters is a result of the learned average energy surface.

The presence of deterministic noise in the DFT/MRCI(2) potentials was dealt with approximately via the inclusion of white noise in the GPR kernel. This leads to a fundamental change in interpretation of the optimized points of (near) degeneracy, and the question of the true existence of conical intersections at the DFT/MRCI(2) level of theory. As a result of the noise term, the surrogate surfaces cannot be made arbitrarily accurate with the addition of more data points, thus affecting the level of degeneracy that can be obtained. Despite the issue with explicitly incorporating noise, however, we have shown that the GPR learning of characteristic polynomial coefficients to account for the deterministic noise present in the DFT/MRCI(2) potentials is a viable method that returns sensible surfaces that optimizes easily with direct optimization methods, and yields MECI geometries in excellent agreement with their ab initio MRCI counterparts.

## Supporting Information Available

Table S1 shows the sample dependence present in both DFT/MRCI(2) and MR-CIS; Tables S2–S4 are the initial geometries used in the DFT/MRCI(2) MECI optimization; Table S5 shows the default SOAP parameters used; Figure S1 shows an overlap of representative DFT/MRCI(2) and MR-CISD MECIs (PDF)
All 50 DFT/MRCI(2) .xyz files of the optimized MECIs for ethylene, budtadiene and fulvene (ZIP)
