# Supplementary material for: Minimum Energy Conical Intersection Optimization Using DFT/MRCI(2)
Source: J Chem Theory Comput. 2025 Jan 29;21(3):1340–52. doi: 10.1021/acs.jctc.4c01489 (PMC11823405; doi:10.1021/acs.jctc.4c01489)
Supplement: Supplementary file 1 — ct4c01489_si_001.pdf [file ct4c01489_si_001.pdf]

Supplementary Information for: Minimum Energy Conical Intersection Optimization Using DFT/MRCI(2)

Tzu Yu Wang,† Simon P. Neville,*,‡ and Michael S. Schuurman*,‡,†

†*Department of Chemistry and Biomolecular Sciences, University of Ottawa, Ottawa, Canada, K1N 6N5*

‡*National Research Council Canada, 100 Sussex Dr., Ottawa, Canada, K1A 0R6*

E-mail: simon.neville@nrc-cnrc.gc.ca; michael.schuurman@uottawa.ca

Sampling

For the MECI optimizations, 3000 structures was generated for the fitting and sampled from bounds of ±0.05 Angstrom per degree of degree of freedom. This was done for all ethylene, butadiene and fulvene For the S_1 optimizations, 3000 structures was generated for the fitting and sampled from bounds of ±0.1 Angstrom per degree of degree of freedom. This was done for both formaldehyde and fulvene

Sampling Error

As mentioned in the main text, the sampling error is present regardless of the electronic structure method. The figure below shows the standard deviations on the optimized kernel hyperparameters for DFT/MRCI(2) and MR-CIS over 50 surrogates with different LHS generated training data. It is clear from the figure that the standard deviation is present for both electronic structure method, and of the same order of magnitude.

Table S1: Surrogate dependence on LHS

	DFT/MRCI	MR-CIS
$\|s_x\|$	±0.011	±0.010
$\|s_y\|$	±0.005	±0.005
$\|g\|$	±0.002	±0.002
$\|h\|$	±0.004	±0.002
$\mathcal{P}$	±0.026	±0.048
$\mathcal{B}$	±0.198	±0.056

Geometries

The nuclear geometries that correspond to the starting guess in the optimization procedure, as well as the all optimized structures, both *ab initio* and GPR-surrogate derived, are presented below.

Initial Geometries

Table S2: Intital ethylene geomery for dftmrci meci optimization

6

C	-0.17298697	0.58333727	0.81200039
C	1.20729392	0.38368139	0.88554229
H	-0.68537087	1.55209287	0.67752414
H	-0.85104543	-0.26356523	0.68856513
H	1.81008863	1.13212229	1.42045414
H	1.27621094	0.95281193	-0.12132797

Table S3: Initital butadiene geometry for dftmrci meci optimization

10

C	1.84796038	0.08270088	0.05844991
C	0.67596898	-0.62977601	-0.02772064
C	-0.5688845	0.0035505	-0.11432183
C	-1.82433999	-0.68278059	0.03936279
H	2.8052618	-0.39894765	0.08356023
H	1.82891932	1.15876798	0.09683816
H	0.68774432	-1.71079421	-0.04068299
H	-0.53087525	1.10166203	-0.13066395
H	-2.67785508	-0.20749418	-0.42651126
H	-1.82849937	-0.02475368	0.96955848

Final Geometries

The set of optimized DFT/MRCI and canonical MR-CIS and MR-CISD MECIS for all the molecule is included in the dftmrci_mecis.zip file and mrci_mecis.zip, respectively.

The overlap of the MECI and S_1 minima structures is shown before in figure **??**, which provides a visual and qualitative comparison. For figure **??**, a representative geometry is chosen based on the R^2 of the fitted surface.

Table S4: Initital Fulvene geometry for dftmrci meci optimization

12

C	-1.68659309e-04	-2.41431930e-05	7.05755656e-01
C	-1.02546406e-04	3.26979258e-06	2.15741154e+00
C	-1.57937548e-04	1.14620206e+00	-1.15542312e-01
C	-7.83792978e-05	-1.14618195e+00	-1.15499289e-01
C	-5.03899948e-05	7.01430377e-01	-1.45013273e+00
C	-3.23621210e-06	-7.01404612e-01	-1.45012716e+00
H	9.22495393e-01	2.01253648e-05	2.72411987e+00
H	-9.22620674e-01	7.95799743e-06	2.72424271e+00
H	-1.72549804e-04	2.16960987e+00	2.32697714e-01
H	-4.43342329e-05	-2.16957998e+00	2.32720667e-01
H	-7.63524246e-06	1.33186308e+00	-2.32451262e+00
H	6.99524859e-05	-1.33183383e+00	-2.32450353e+00

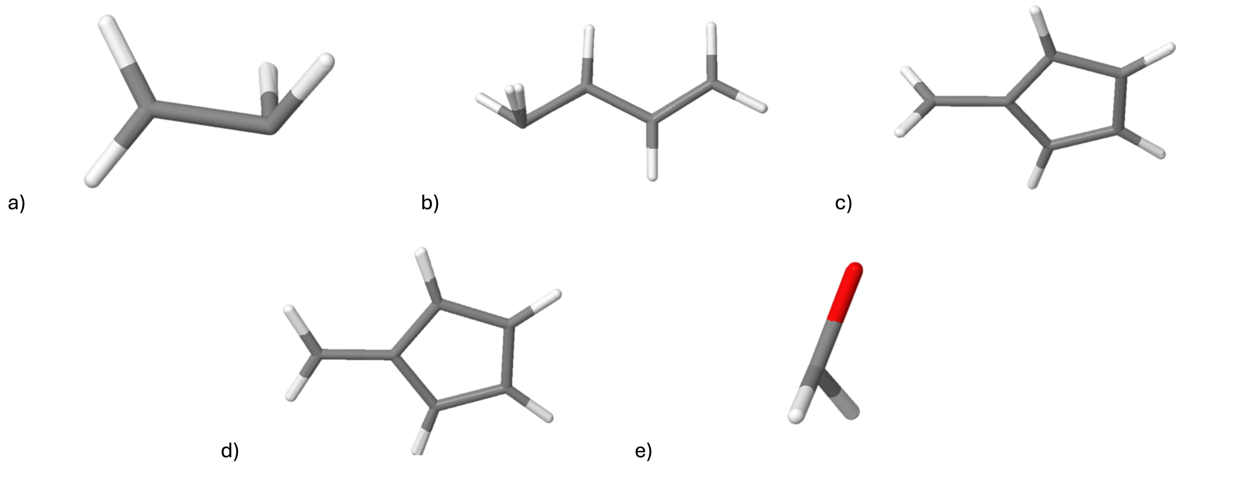

Figure S1: Overlap of the DFT/MRCI and MR-CISD MECI and S_1 minimum structures.

SOAP parameters

The SOAP power spectrum for this work was computed using dscribe.[?, ?]

Table S5: Default SOAP parameters used

r_{cut}	4
n	6
l	6
σ	0.1
